# Liposome Model Systems to Study the Endosomal Escape of Cell-Penetrating Peptides: Transport across Phospholipid Membranes Induced by a Proton Gradient

**DOI:** 10.1155/2011/897592

**Published:** 2010-12-28

**Authors:** Fatemeh Madani, Alex Perálvarez-Marín, Astrid Gräslund

**Affiliations:** ^1^Department of Biochemistry and Biophysics, Arrhenius Laboratories for Natural Sciences, Stockholm University, 10691 Stockholm, Sweden; ^2^Department of Anesthesia, Brigham and Women's Hospital, Boston, MA 02115, USA; ^3^Centre d'Estudis Biofísics, Universitat Autònoma de Barcelona, 08193 Bellaterra, Spain

## Abstract

Detergent-mediated reconstitution of bacteriorhodopsin (BR) into large unilamellar vesicles (LUVs) was investigated, and the effects were carefully characterized for every step of the procedure. LUVs were prepared by the extrusion method, and their size and stability were examined by dynamic light scattering. BR was incorporated into the LUVs using the detergent-mediated reconstitution method and octyl glucoside (OG) as detergent. The result of measuring pH outside the LUVs suggested that in the presence of light, BR pumps protons from the outside to the inside of the LUVs, creating acidic pH inside the vesicles. LUVs with 20% negatively charged headgroups were used to model endosomes with BR incorporated into the membrane. The fluorescein-labeled cell-penetrating peptide penetratin was entrapped inside these BR-containing LUVs. The light-induced proton pumping activity of BR has allowed us to observe the translocation of fluorescein-labeled penetratin across the vesicle membrane.

## 1. Introduction

Live cells are protected from the surrounding environment by the cell membrane, which only allows compounds with a small molecular size to pass this barrier into the cell. Some drug molecules, on the other hand, are large hydrophilic molecules, and this creates major limitations for their penetration through the cell membrane. With discovery of cell-penetrating peptides (CPPs), the transport of such molecules can be accomplished. Generally, CPPs are defined as short, water soluble and partly hydrophobic and/or polybasic peptides (at most 30–35 amino acid residues) with a net positive charge at physiological pH [1]. This new class of peptides was introduced in the late 1980s by the discovery of the human immunodeficiency virus type 1 (HIV-1) encoded Tat peptide [2, 3] and the amphiphilic Drosophila Antennapedia homeodomain-derived 16 amino acid penetratin peptide (pAntp), which was discovered somewhat later [4–7]. These two peptides are the most extensively studied of all CPPs. 

The main feature of CPPs is that they are able to penetrate the cell membrane at low micromolar concentrations *in vivo* and *in vitro* without using any chiral receptors and without causing irreversible membrane damage. These peptides are capable of internalizing electrostatically or covalently bound biologically active cargoes such as drugs, with high efficiency and low toxicity [1, 8].

Despite many studies made on CPPs, the mechanism(s) by which CPPs enter the cells has not been completely resolved. There is some evidence for both energy-independent processes and endocytosis in internalization of CPPs. Presently, endocytosis, composed of two steps, endocytotic entry followed by endosomal escape, is believed to be the most common uptake mechanism at low CPP concentrations [8, 9]. 

Model biomembranes or lipid bilayers are efficient model systems to investigate the CPPs translocation mechanism(s). Large unilamellar vesicles (LUVs) are among the most commonly used model membranes in lipid-peptide interaction studies [10].

Here, we have performed experiments to study the background mechanism(s) of endosomal escape. Cell membranes are normally weakly negatively charged and consist of different phospholipid molecules and associated proteins and proteoglycans. The lipids used in our study (a mixture of zwitterionic POPC and negatively charged POPG phospholipids) have been chosen to mimic cell membranes. Bacteriorhodopsin (BR) reconstituted into LUVs with 20% negatively charged phospholipid are used to model the endosomes. The LUVs were prepared by the extrusion method, and their size and stability were carefully examined by dynamic light scattering (DLS). 

BR is an integral membrane protein of about 26 KDa found in *Halobacterium salinarium.* There are various methods to reconstitute membrane proteins into the vesicles including organic solvent-mediated reconstitution, direct incorporation into preformed liposomes, mechanical means, and the detergent-mediated reconstitution method. Among these methods, detergent-mediated reconstitution is the most common and successful technique to incorporate membrane proteins into vesicles [11]. The final orientation of the protein incorporated into the vesicle bilayers depends on several factors; one of the most critical is the detergent composition in the proteoliposomes [12]. When BR absorbs light, it pumps protons in a direction that depends on the direction of protein insertion into the membrane and generates an H^+^ gradient and membrane potential [13]. The detergent-mediated reconstitution method can provide 95% inside-out orientation of BR in the bilayer indicating that BR pumps protons from the outside to the inside of vesicles [11]. In the following, some practical aspects crucial for the reproducibility of the method are described. Furthermore, we have studied the translocation ability of fluorescein-labeled penetratin in the presence of a pH gradient across an LUV membrane.

## 2. Materials and Methods

### 2.1. Materials

1-palmitoyl-2-oleoyl-*sn*-glycero-3-phospho-choline (POPC) and 1-palmitoyl-2-oleoyl-*sn*-glycero-3[phospho-rac-(1-glycerol)] (POPG) used in this study were obtained from Avanti Polar Lipids (Alabaster, Alabama, USA) and were used without any extra purification. The detergent n-octyl-*β*-D-glucopyranoside (OG) was from Glycon Biochemicals (Luckenwalde, Germany). PD-10 desalting columns were purchased from GE Healthcare (Buckinghamshire, UK). Bio-Beads were from BIO-RAD (California, USA). Fluorescein-labeled penetratin was produced by Neosystem Laboratories (Strasbourg, France). *Halobacterium salinarum* strain S9 was a generous gift from Professor Esteve Padrós (Universitat Autonoma de Barcelona, Spain). Bacteriorhodopsin (BR) was produced and purified essentially according to a published protocol [14]. A UV-Vis absorption spectrum of the purified BR was recorded within the 800–250 nm range to check the purity of the sample and to calculate the concentration (*ε* = 62700 M^−1^ cm^−1^ at 568 nm, MW = 26000 Da). Aliquots at the desired concentration were stored at −20°C.

### 2.2. Vesicle Preparation

The extrusion method is a common method for vesicle preparation, which produces LUVs with a narrow size distribution [15]. We used a hand-driven extrusion apparatus with one milliliter capacity. In this method, 20% negatively charged LUVs are prepared by dissolving the lipids (neutral POPC and negatively charged POPG) at the total concentration of 20 mM in chloroform to obtain a homogeneous mixture of the lipids. Then, the solvent is removed by evaporation under high vacuum for 3 hr. The resulting dried lipid film is resuspended by adding a buffer solution (20 mM phosphate buffer, 100 mM KCl, pH 7.2). This liposomal suspension is then vortexed for 10 minutes followed by 5 freeze-thaw cycles to reduce the lamellarity and obtain more aqueous trapped volumes. After the freezing and thawing cycles, the lipid suspension containing multilamellar vesicles is pushed through two polycarbonate filters (100 nm pore size) 20 times by using an Avanti manual extruder. This results in LUVs with a well-defined and homogeneous size.

### 2.3. Reconstitution of BR into LUVs: Detergent-Mediated Reconstitution Method

The preparation of BR-reconstituted LUVs consists of three steps: vesicle solubilization, BR addition, and detergent removal [11, 12, 16].

#### 2.3.1. Vesicle Solubilization

**Figure 1:**
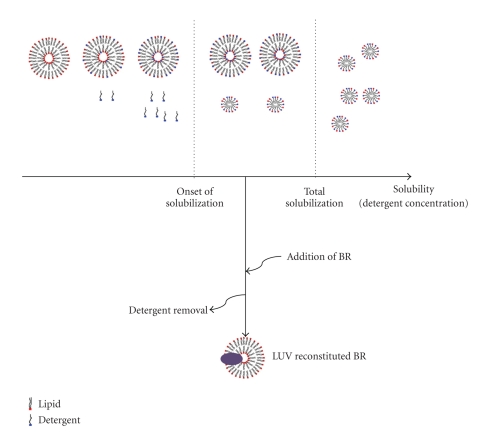
Scheme for the detergent-mediated reconstitution of BR into LUVs (after [11]). Stage I–III: Gradual addition of detergent to LUVs. For optimal reconstitution efficiency, BR should be added during stage II. Detergent is removed by Bio-Beads, and the result is an LUV with incorporated BR.

LUVs prepared by extrusion method were diluted in the buffer used for their preparation to the desired concentration. Here, we have used 2.3 mL vesicle suspension of 5 mM phospholipid concentration. After the addition of the detergent, LUV solubilization takes place in three stages (Figure 1); first the detergent monomers diffuse among bilayers, and at the same time there are some free detergent monomers in the solution (stage I). The permeability, size, and stability of the LUVs will change. Further addition of detergent saturates the vesicle bilayer. At stage II, when free detergent monomer concentration reaches its cmc value, transition from monomers to mixed lipid/detergent micelles will occur. At this step, both saturated vesicles and mixed micelles coexist. Stage III is the point where all LUVs have disappeared and only mixed micelles are present in the solution.

The choice of detergent and its concentration affect this three-stage mechanism. In the present paper, octyl glucoside (OG) has been used. OG is a nonionic detergent with a cmc value of about 25 mM that facilitates its removal [17]. Here, after adding OG, the final concentrations of lipid and OG were 4.8 mM and 25.6 mM, respectively.

#### 2.3.2. BR Addition

After 5–10 min of the vesicle solubilization, BR monomers resulting from detergent solubilization of purple membrane (BR 1 mg/mL, OG 100 mM) were added to the solubilized LUVs suspension and incubated for 5 to 10 minutes. The resulting suspension should be a mixture of BR/lipid/detergent vesicles and lipid/detergent micelles with the final concentrations of 4 *μ*M, 4.3 mM, and 29 mM for BR, lipid, and detergent, respectively. At this stage, BR may be incorporated into the vesicles which have been saturated and destabilized by the detergent. As suggested also in [11], by varying the detergent/lipid ratio in the BR incorporation process, we found that the partly detergent-saturated LUVs are optimal in reconstitution of BR. The detergent-BR-phospholipid mixtures were kept at room temperature for 5 min to 15 min, and the detergent was then removed.

#### 2.3.3. Detergent Removal

The method of detergent removal highly affects the results of the reconstitution process. High proton pumping activity of BR-reconstituted vesicles requires sealed vesicles which result from removing all residual detergents from the suspension. Any remaining detergent may alter the size, permeability, and stability of the vesicles produced by detergent removal from mixed micelles. In addition, the rate of detergent removal is another factor affecting the reconstitution process. It was observed that both initial rates and total amounts of H^+^ pumping decrease when the rate of detergent removal increases [12, 16].

There are several strategies to remove detergent from mixed lipid/protein/detergent vesicles. The nature of the detergent affects the method that has to be employed. Bio-Beads can absorb almost any kind of detergents with a wide range of cmc values. For example, Triton-X with a low cmc value cannot be easily removed by the dialysis method. However, absorption by hydrophobic Bio-Beads may efficiently remove even low cmc value detergents [18].

Detergent removal should best be performed in two steps: first wet Bio-Beads (80 mg/mL) were directly added to the BR-lipid-detergent suspension. The mixture was lightly stirred at room temperature. Transition from micellar to lamellar may take place at this stage. After 3 hr of incubation at room temperature, a second portion of slightly wet beads was added and mixed overnight with a small shaker and the rate of around 400 rpm to remove residual detergents. At the end, two PD-10 columns were used to remove Bio-Beads and residual detergents from the sample.

### 2.4. pH Measurement

In order to monitor the pH changes outside the vesicle, we prepared an experiment using a Xenon lamp to illuminate the sample and the pH meter (PHM 93 Reference pH meter and Thermo Scientific model 320 electrode) to record the values of the pH. The BR-reconstituted vesicle suspension was equilibrated in 120 mM KCl pH 7.4 buffer using a PD-10 column. The BR-sample was kept in the dark at least 30 min to ensure the dark adaptation of the sample, and the pH was recorded in the dark as the baseline. Light-induced pH changes of BR-reconstituted LUVs were measured in a cuvette under agitation.

### 2.5. Preparation of CPPs-Entrapping LUVs

A 20 *μ*M fluorescein-labeled penetratin solution was prepared in 20 mM potassium phosphate, 100 mM KCl (pH 7.2), and 100 mM potassium iodide (KI) used as a quencher. LUVs containing the peptide were prepared as described earlier by using this solution as buffer. At this stage, BR may be introduced into the LUVs according to the procedure described above. Finally, the LUV suspension was washed twice using two PD-10 columns to remove non-encapsulated fluorescein-labeled penetratin and quencher from the outside of the LUVs. It is important to remove components outside the vesicles (e.g., peptides or quencher) after the detergent removal stage since detergent changes the membrane permeability, and it is not worth removing them before this stage. KI was used to quench and minimize the background fluorescence intensity. Thus, any increase in background fluorescence is due to the leakage of the labeled peptide from the LUVs. 

At the end of this preparation, the sample had a total lipid concentration estimated to about 2.3 mM. Based on vesicle geometry (diameter 100 nm) each vesicle contained about 10^5^ lipids. This would yield an approximate vesicle concentration of 2.3 ∗ 10^−8^ M. In BR-containing vesicles, the BR concentration in the sample was estimated by measuring the light absorption after treatment with Triton-X, using the *ε* of 62700 M^−1^ cm^−1^ at 568 nm. The concentration of BR was found to be around 1 *μ*M corresponding to about 40 BR molecules inserted per vesicle.

### 2.6. Fluorescence Spectroscopy and CPP Leakage Study

To study the effect of a pH gradient on the CPP escape from LUVs, we used fluorescence spectroscopy. Fluorescence was measured in a Horiba Jobin Yvon Fluorolog-3 spectrometer using the DataMax operating software and with a 4 ∗ 10 mm quartz cuvette. The sample was excited at 494 nm, and its emission was scanned from 505 to 550 nm with 1 nm emission and excitation bandwidths. All experiments were run at 20°C.

### 2.7. Circular Dichroism (CD) Spectroscopy

CD was used to determine the secondary structure of the BR reconstituted in the vesicles. CD spectra were recorded on a Chirascan CD spectrometer at 20°C. Wavelengths between 190 nm and 260 nm were recorded, using a bandwidth of 2 nm. A quartz cuvette with an optical path length of 2 mm was used, requiring approximately 500 *μ*L of sample. The temperature was adjusted using a TC 125 temperature control. The background spectra of the vesicle solution were subtracted from the peptide spectra. Spectra were collected and averaged over ten measurements.

### 2.8. Dynamic Light Scattering (DLS)

DLS was used to determine the hydrodynamic radius of the vesicle and BR-reconstituted vesicles. Measurements were carried out using a light scattering instrument ALV/CGS-3 equipped with a Light Scattering Electronics and Multiple Tau Digital correlator ALV/LSE-5004. Correlation data were acquired typically for 3 runs each for 30 sec. Correlation functions at 150° were recorded at the temperature of 20°C using a Julabo temperature control. The hydrodynamic radius was calculated using the ALV software, unweighted fitting.

## 3. Results and Discussion

**Figure 2:**
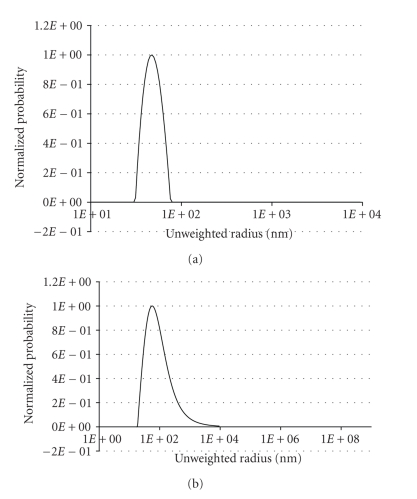
Unweighted size distribution for (a) LUVs and (b) BR-reconstituted LUVs.

We prepared 20% negatively charged LUVs composed of 80% POPC and 20% POPG by the extrusion technique. BR was reconstituted into the LUVs using the detergent-mediated reconstitution method. The resulting LUVs without and with BR were characterized using dynamic light scattering. As shown in Figures 2(a) and 2(b), both samples have a relatively homogenous population with slightly different vesicle sizes. According to the detergent-mediated reconstitution method [11], BR is oriented in the membrane of LUVs such that it pumps protons from the outside to the inside of the vesicles upon illumination. 

**Figure 3:**
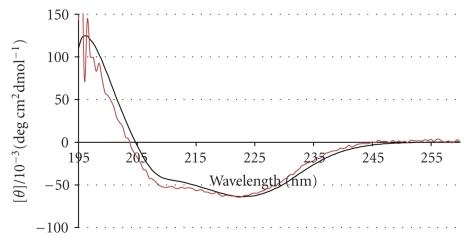
Circular dichroism spectra of 2 *μ*M BR in 100 mM OG detergent (black) and reconstituted LUVs (red) at 20°C.

In addition, we used CD spectroscopy to determine whether the reconstitution process affected the secondary structure of BR (Figure 3). The secondary structure of BR was dominated by *α*-helix both in free solution and when reconstituted into the LUVs.

**Figure 4:**
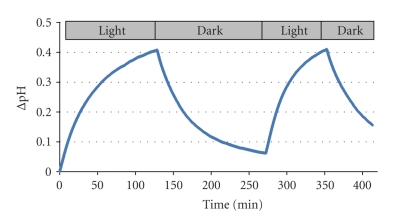
pH changes outside the BR-reconstituted vesicle as a function of illumination time. Conditions: 20 mM potassium phosphate buffer and 100 mM KCl, pH 7.2 inside the 20% negatively charged LUVs, 120 mM KCl outside the LUVs, pH = 7.4, 25°C.

Upon illumination of the sample, the measured pH increased and reached a maximum on the outside of the LUVs (Figure 4) indicating that most of the BR molecules are properly oriented towards the interior of the vesicles. Translocation of protons from the outside to the inside vesicles resulted in a more acidic pH inside the LUVs. When illumination was discontinued, the measured pH outside the LUVs decreased, indicating that protons leaked out again across the membrane and reached an equilibrium (Figure 4). As shown in this figure, the proton pumping process can be repeated with the same sample.

We have repeated the experiment in the absence of BR to investigate whether this effect observed is due to the proton pumping of BR or some other effects. No changes in pH were observed upon illumination of LUVs in the absence of BR which indicates that light-induced pH changes are indeed due to the proton pumping of BR (data not shown).

The change in pH (ΔpH) outside the vesicles can be used to calculate the corresponding ΔpH inside the vesicles based on proton concentration and the estimated inner volume of all vesicles in the solution. A ΔpH outside the vesicles of +0.2 after 25 min corresponds to almost −2 pH units inside the vesicles under the conditions used here. 

**Figure 5:**
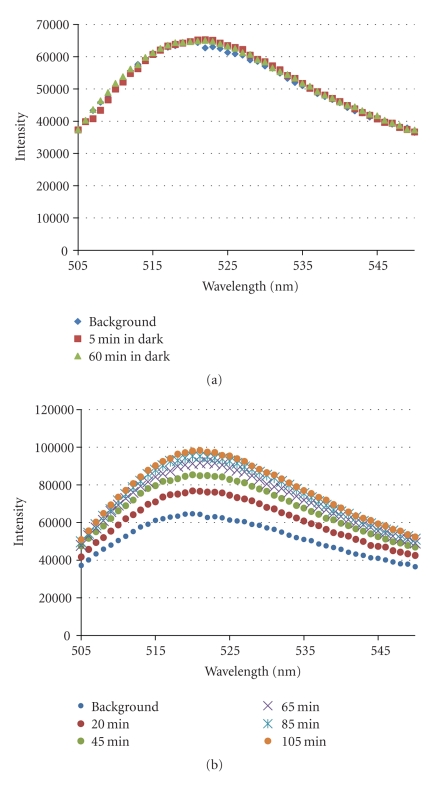
Fluorescence changes of the sample containing BR-LUVs with fluorescein-labeled penetratin and fluorescence quencher KI inside the vesicles. Changes in fluorescence intensity between 505 and 550 nm (excitation wavelength 494 nm) were recorded in the (a) absence of illumination and (b) in the presence of light for indicated periods of time. Conditions: 20 mM potassium phosphate, 100 mM KCl buffer, 20 *μ*M fluorescein-labeled penetratin, and 100 mM KI inside the LUVs, total lipid concentration 2.3 mM, pH 7.4, and 20°C.

We also evaluated the effect of the pH gradient on the translocation abilities of the fluorescein-labeled CPP penetratin. BR with the inside-out orientation was reconstituted into LUVs. Upon illumination, BR pumps protons into the LUVs creating a pH gradient over the membrane. Fluorescein-labeled penetratin together with KI as a quencher was enclosed in the BR-reconstituted LUVs.  Figure 5 shows the fluorescence intensity changes of the sample containing BR-reconstituted LUVs and fluorescein-labeled penetratin together with fluorescence quencher KI inside the LUVs. 

In the dark, we observed no changes in the fluorescence intensity, indicating insignificant leakage of the peptide out of the LUVs. The peptides are not able to translocate across the membrane without any promoting proton gradient (Figure 5(a)).

Efficient peptide escape was observed in the presence of the light. A significant increase in the fluorescence intensity was observed when the sample was illuminated. This result indicates that a pH gradient across the membrane enhances the vesicular escape for the examined fluorescein-labeled CPP (Figure 5(b)). 

Longer period of illumination leads to more leakage of the CPP. However, after around 100 min, it reaches an almost stable condition, corresponding to the transport of around 30% of the fluorescein-labeled penetratin out of the LUVs (data not shown). In this experiment, a 100% release was achieved when the fluorescence intensity was measured after addition of 10% (w/v) Triton X-100 detergent.

## 4. Conclusions

Our studies show that there are several factors affecting the result of the proton pumping experiment, starting from vesicle preparation to the detergent removal. The most important of these factors concerns the permeability and stability of the LUVs which strongly affects the proton pumping activity and hence pH gradient of the resulting BR-vesicles. Leaking vesicles display lower pH gradient due to the proton leakage from the membrane. Further, it is important to use lipids with high purity and to ensure complete removal of the detergent. Finally, one should examine the vesicles by DLS to verify their homogeneity and size.

The degree of orientation of the BR incorporated into the LUVs also affects the proton pumping efficiency. It has been shown that 95% inside-out orientation will be achieved using the detergent-mediated reconstitution method. However, this percentage strongly depends on the experimental conditions, for example, detergent to lipid ratio and the time point, where BR will be added to the LUVs [11].

Overall, our observations are in agreement with the earlier preliminary results with labeled penetratin by Björklund et al. [19]. 

Use of an ionophore nigericin is another alternative to create acidic pH inside the vesicles [20]. It works by exchanging K^+^ for H^+^ across the vesicle membrane and creating a transmembrane pH gradient. However, the effect of nigericin is dependent on the presence of high concentrations of a K^+^ salt inside the vesicles. To create a transmembrane salt gradient, metal ions have to be removed from outside the vesicles by passing through the columns equilibrated by high concentrations of, for example, sucrose. High-concentrated sugar and metal ions may destabilize the vesicles resulting in leakage of the protons and hence decreasing the pH gradient. 

The light-induced BR proton pumping experiment has the advantages that (1) it does not require any special buffer which alters the vesicle stability, (2) one is able to control pumping activity by the illumination time period, and (3) several experiments can be carried out with the same sample repeating dark-illumination cycles. The present studies also suggest a general mechanism by which positively charged molecules, other than peptides, may enter into cells by endocytotic uptake followed by escape from the acidified endosome.
